# Supplementary material for: A yeast phenomic model for the influence of Warburg metabolism on genetic buffering of doxorubicin
Source: Cancer Metab. 2019 Oct 23;7:9. doi: 10.1186/s40170-019-0201-3 (PMC6806529; doi:10.1186/s40170-019-0201-3)
Supplement: Supplementary file 9 — Additional file 9. GO term-specific heatmaps for REMc/GTF-enriched clusters. GO term-specific heatmaps for significant GO process terms were generated as described in methods and Figs. 3 and 4. Any related child terms are presented in subsequent pages of the parent file name. GO terms with more than 100 children, with 2 or fewer genes annotated to the term, or a file size over 300KB are not shown. All heatmaps are generated with the same layout (see Figs. 3 and 4). [file 40170_2019_201_MOESM9_ESM.bz2 › Additional_File9_GOTermHeatmaps/Additional_File9_GOTermHeatmap/tRNA_aminoacylation.pdf]

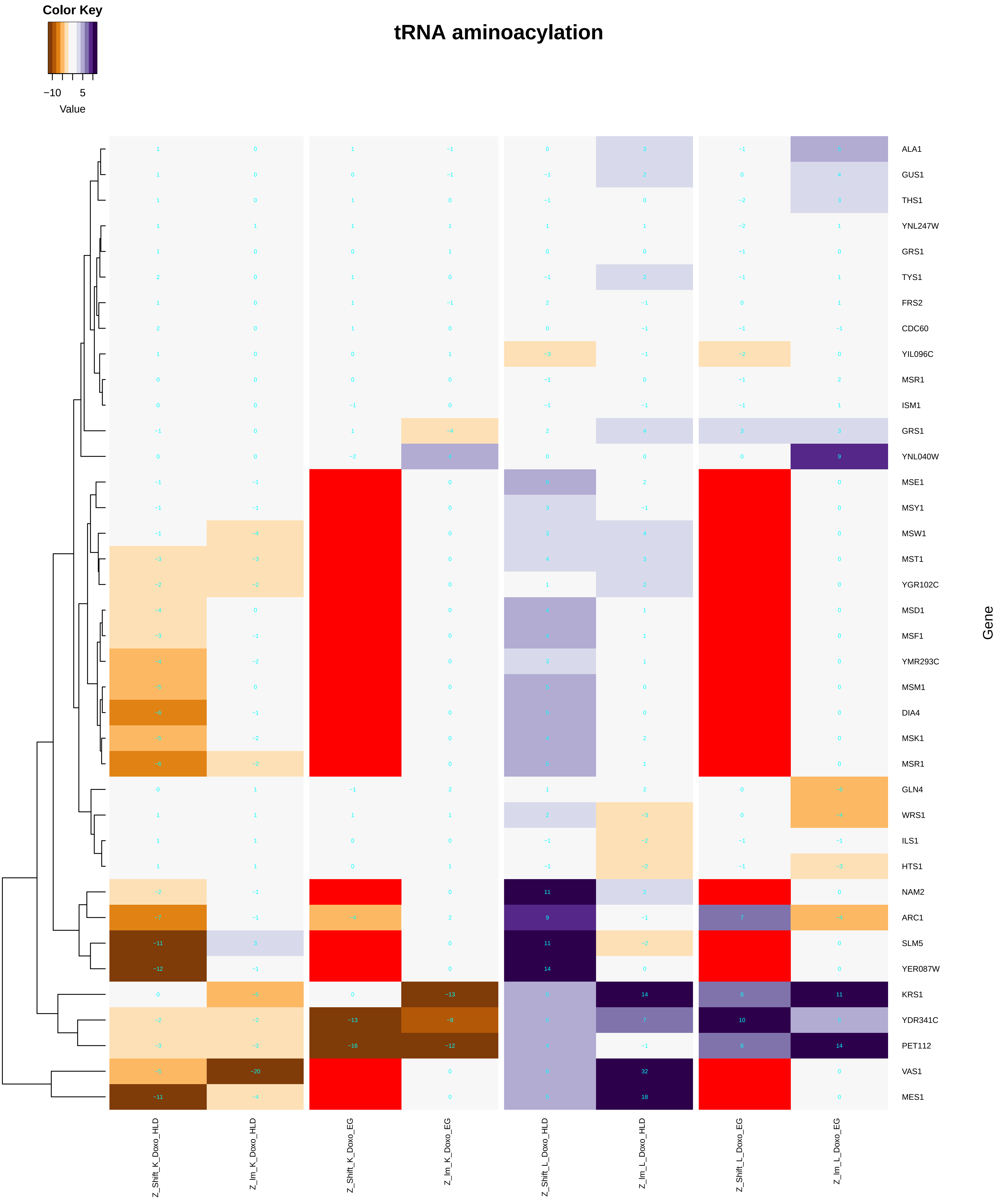
tRNA aminoacylation
Color Key
−10
5
Value
ALA1
GUS1
THS1
YNL247W
GRS1
TYS1
FRS2
CDC60
YIL096C
MSR1
ISM1
GRS1
YNL040W
MSE1
MSY1
MSW1
MST1
YGR102C
MSD1
MSF1
YMR293C
MSM1
DIA4
MSK1
MSR1
GLN4
WRS1
ILS1
HTS1
NAM2
ARC1
SLM5
YER087W
KRS1
YDR341C
PET112
VAS1
MES1
Gene
Z_Shift_K_Doxo_HLD
Z_lm_K_Doxo_HLD
Z_Shift_K_Doxo_EG
Z_lm_K_Doxo_EG
Z_Shift_L_Doxo_HLD
Z_lm_L_Doxo_HLD
Z_Shift_L_Doxo_EG
Z_lm_L_Doxo_EG

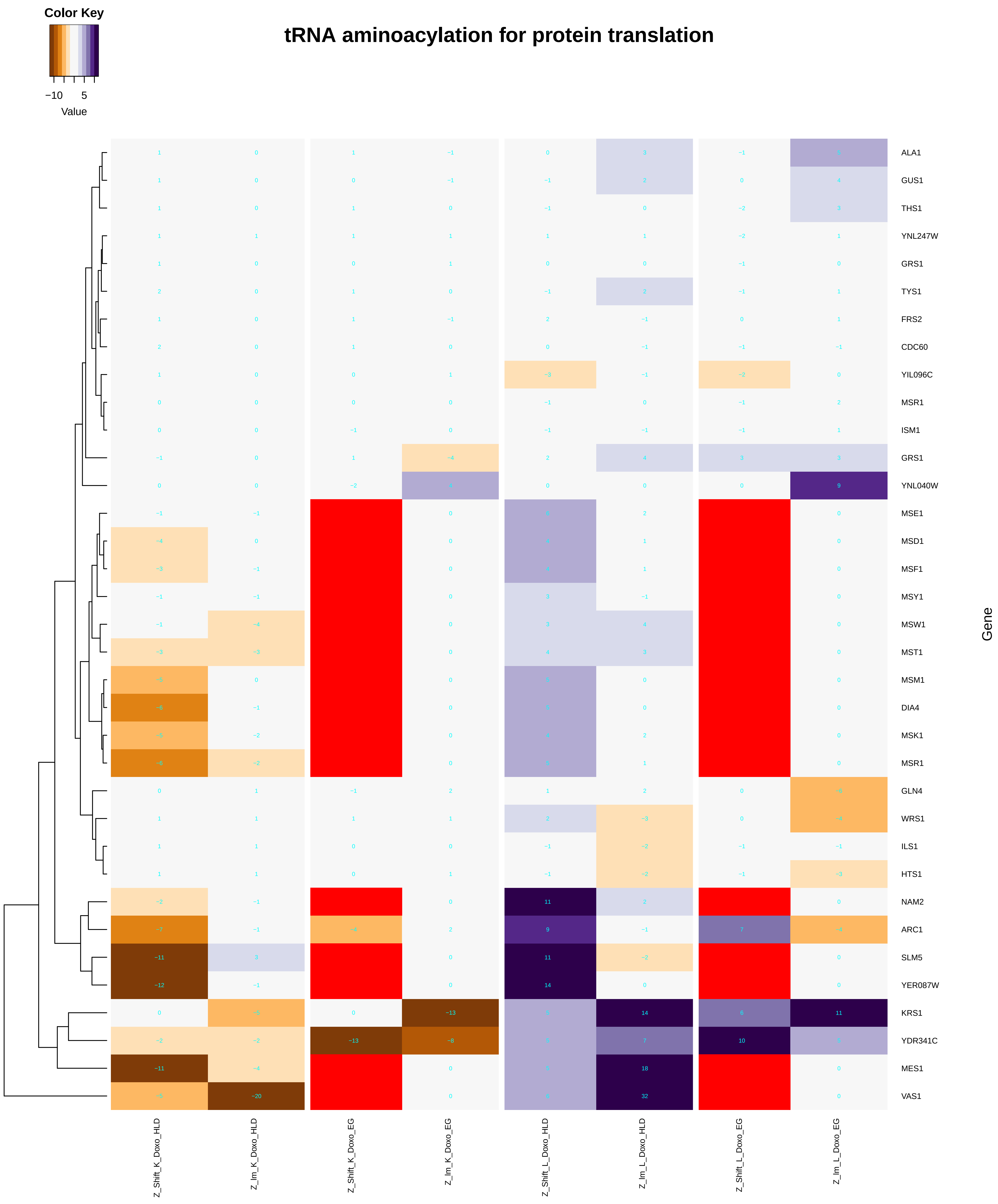
Color Key
−10 5
Value
tRNA aminoacylation for protein translation
ALA1
GUS1
THS1
YNL247W
GRS1
TYS1
FRS2
CDC60
YIL096C
MSR1
ISM1
GRS1
YNL040W
MSE1
MSD1
MSF1
MSY1
MSW1
MST1
MSM1
DIA4
MSK1
MSR1
GLN4
WRS1
ILS1
HTS1
NAM2
ARC1
SLM5
YER087W
KRS1
YDR341C
MES1
VAS1
Gene
Z_Shift_K_Doxo_HLD
Z_lm_K_Doxo_HLD
Z_Shift_K_Doxo_EG
Z_lm_K_Doxo_EG
Z_Shift_L_Doxo_HLD
Z_lm_L_Doxo_HLD
Z_Shift_L_Doxo_EG
Z_lm_L_Doxo_EG

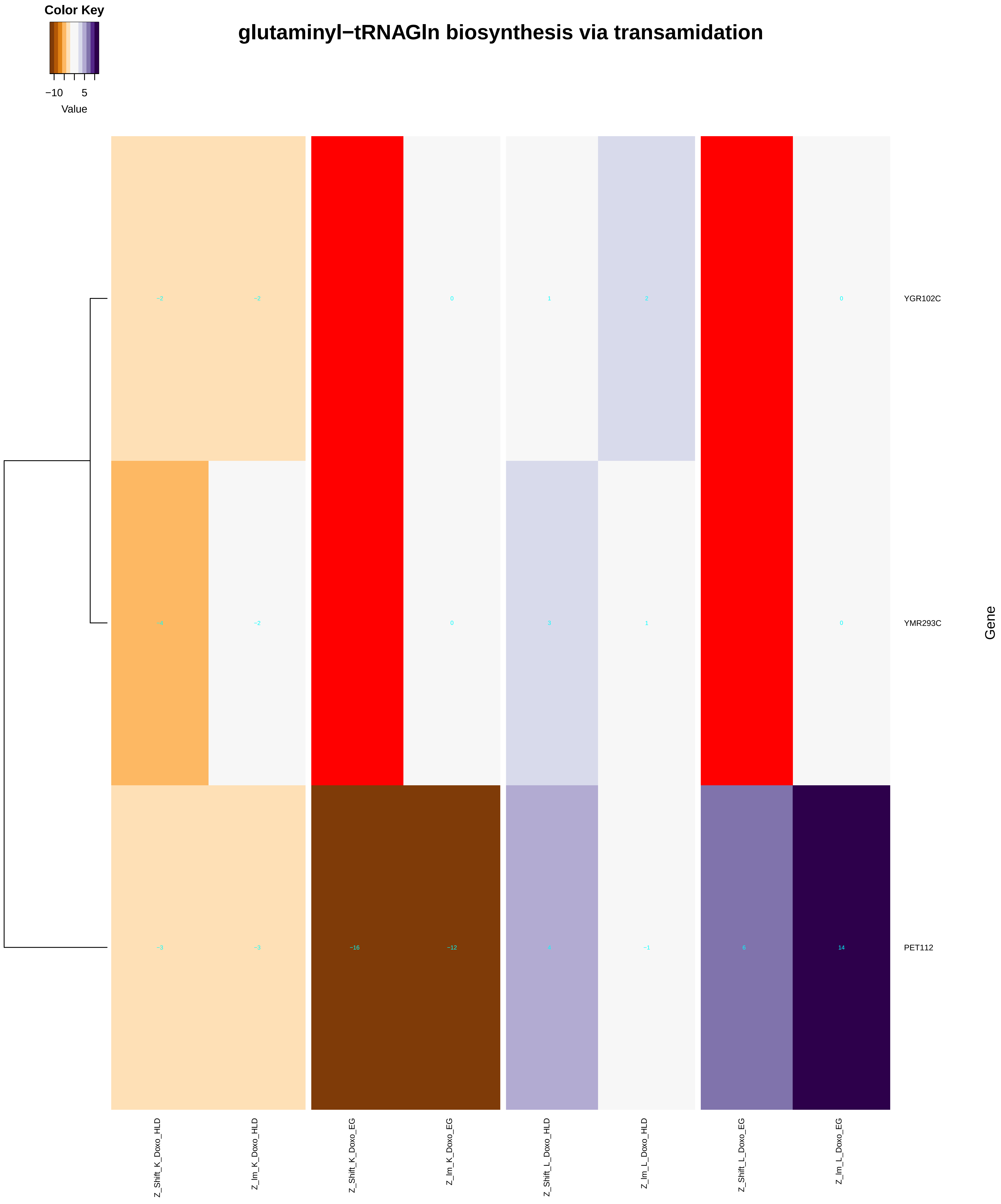

glutaminyl−tRNAGln biosynthesis via transamidation
Color Key
−10
5
Value
YGR102C
YMR293C
PET112
Gene
Z_Shift_K_Doxo_HLD
Z_lm_K_Doxo_HLD
Z_Shift_K_Doxo_EG
Z_lm_K_Doxo_EG
Z_Shift_L_Doxo_HLD
Z_lm_L_Doxo_HLD
Z_Shift_L_Doxo_EG
Z_lm_L_Doxo_EG

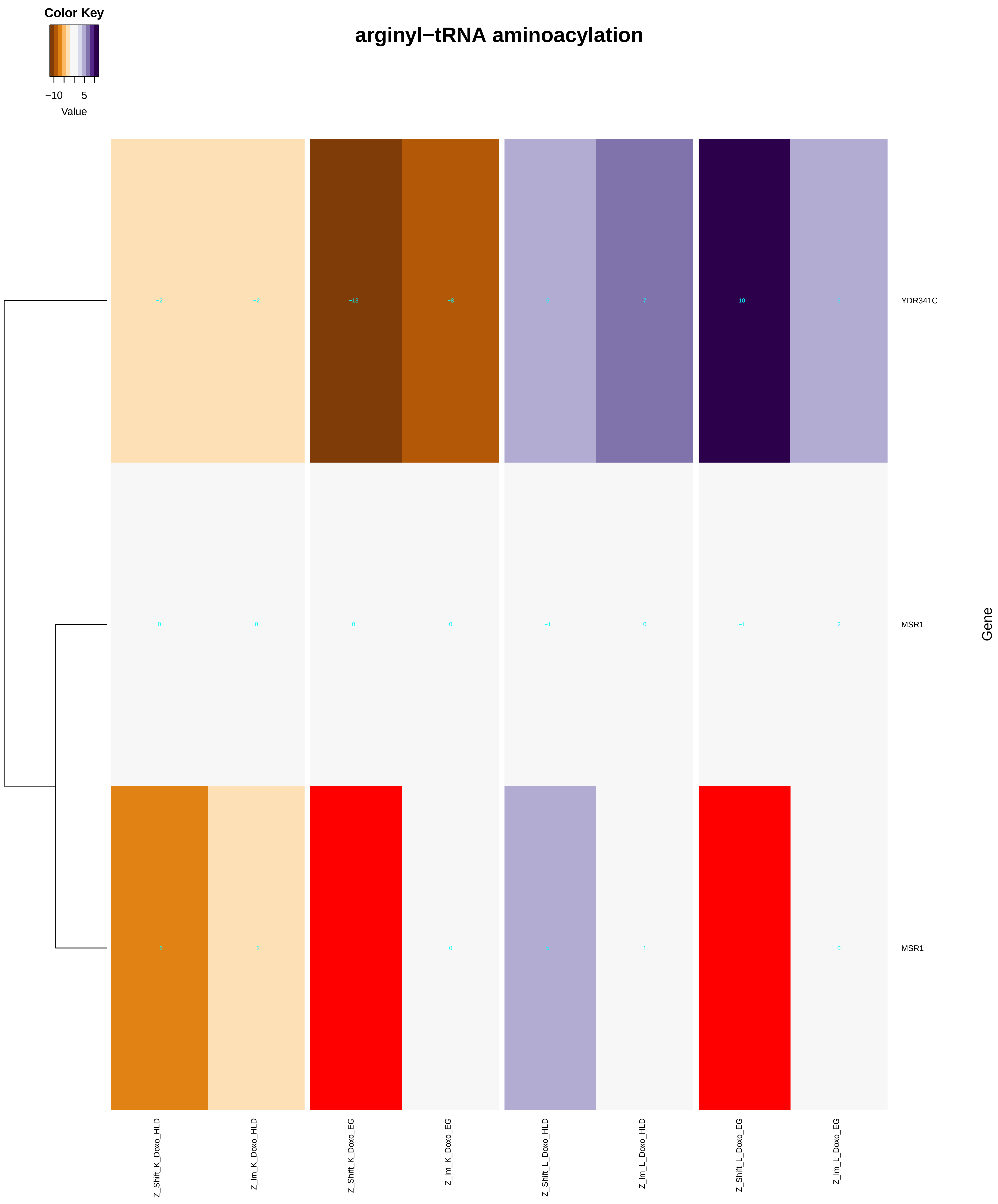
Color Key
−10
5
Value
arginyl−tRNA aminoacylation
YDR341C
MSR1
MSR1
Gene
Z_Shift_K_Doxo_HLD
Z_Im_K_Doxo_HLD
Z_Shift_K_Doxo_EG
Z_Im_K_Doxo_EG
Z_Shift_L_Doxo_HLD
Z_Im_L_Doxo_HLD
Z_Shift_L_Doxo_EG
Z_Im_L_Doxo_EG

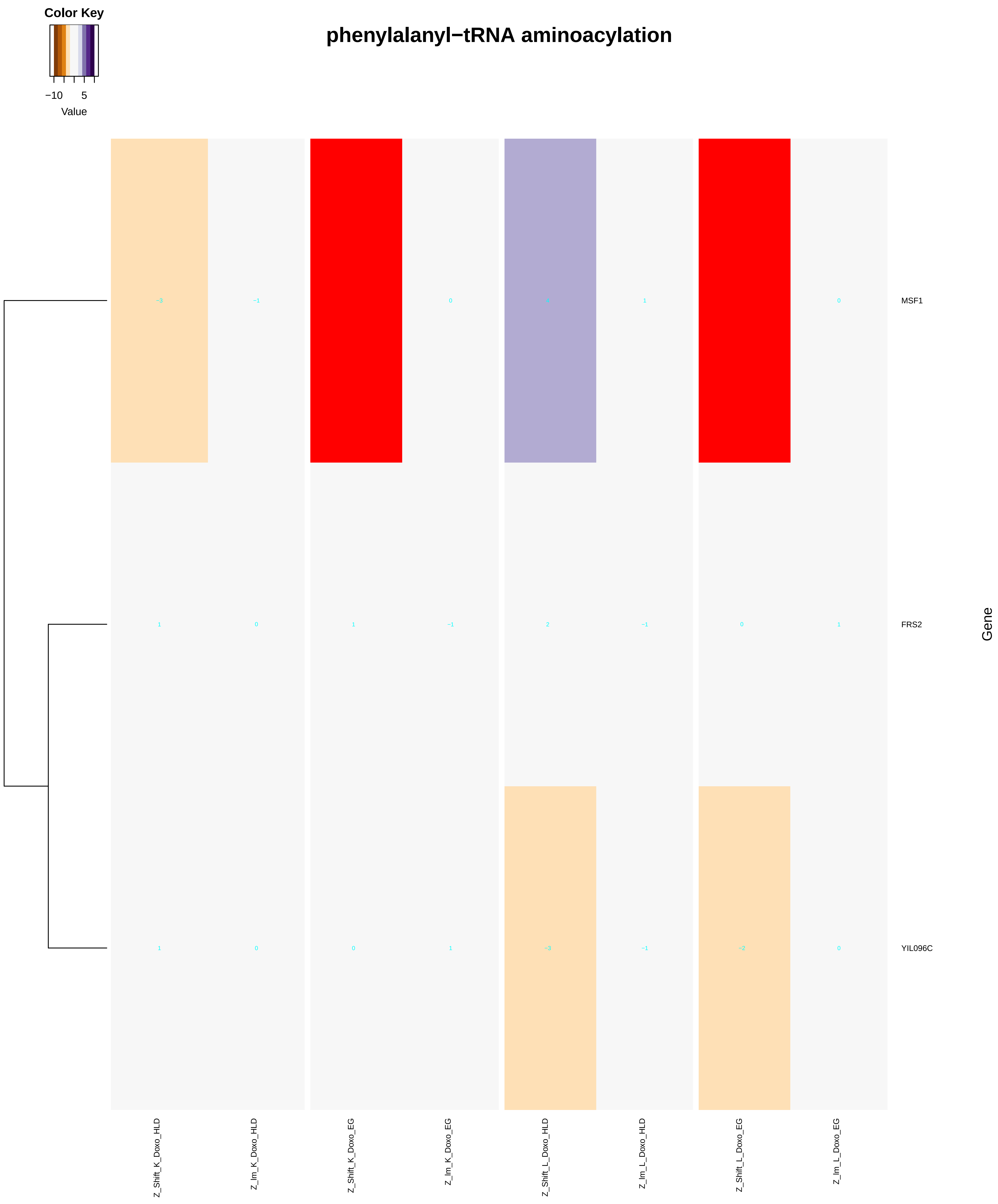

phenylalanyl−tRNA aminoacylation
Color Key
−10
5
Value
MSF1
FRS2
YIL096C
Gene
Z_Shift_K_Doxo_HLD
Z_lm_K_Doxo_HLD
Z_Shift_K_Doxo_EG
Z_lm_K_Doxo_EG
Z_Shift_L_Doxo_HLD
Z_lm_L_Doxo_HLD
Z_Shift_L_Doxo_EG
Z_lm_L_Doxo_EG

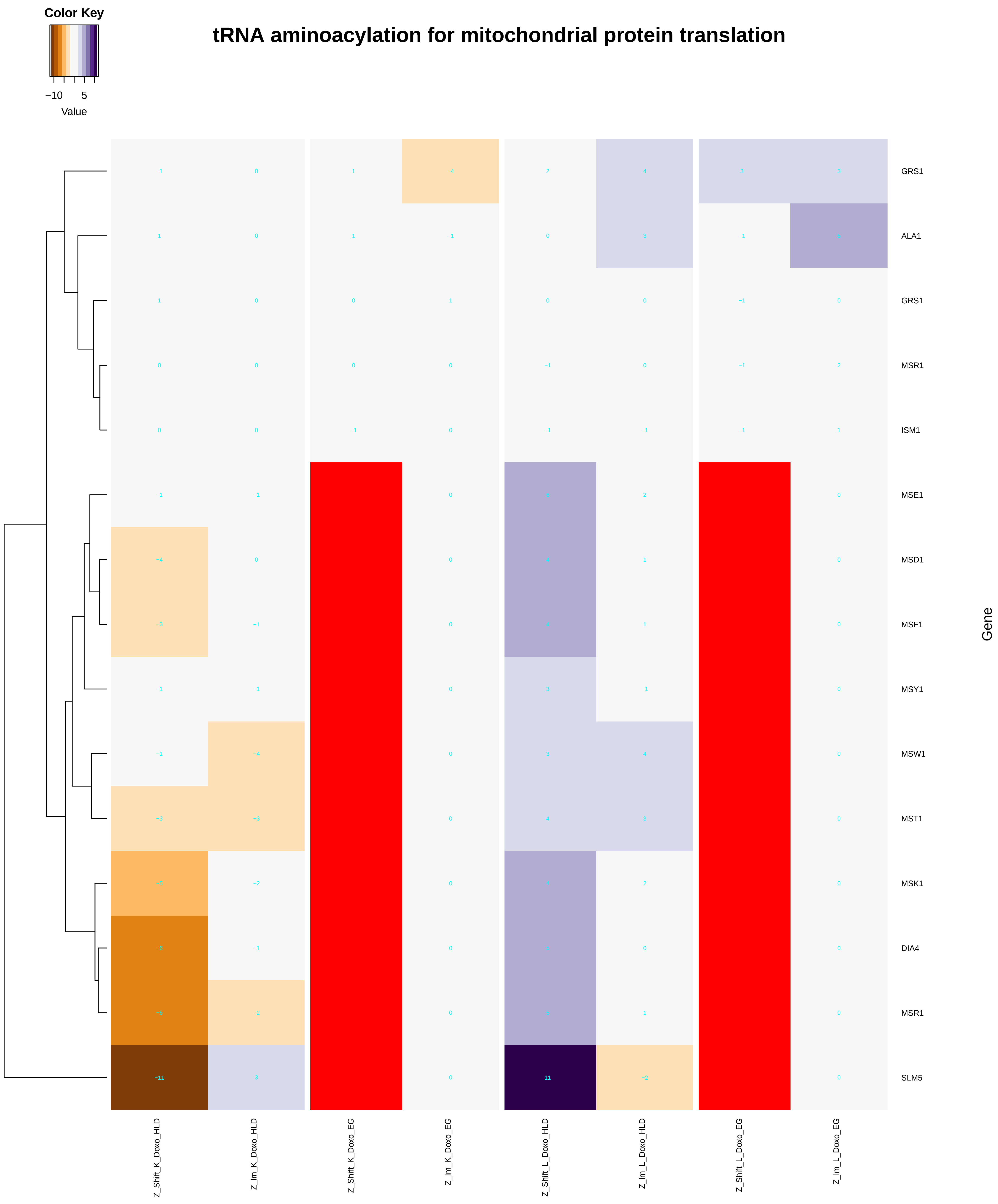
tRNA aminoacylation for mitochondrial protein translation
Color Key
−10
5
Value
GRS1
ALA1
GRS1
MSR1
ISM1
MSE1
MSD1
MSF1
MSY1
MSW1
MST1
MSK1
DIA4
MSR1
SLM5
Gene
Z_Shift_K_Doxo_HLD
Z_Im_K_Doxo_HLD
Z_Shift_K_Doxo_EG
Z_Im_K_Doxo_EG
Z_Shift_L_Doxo_HLD
Z_Im_L_Doxo_HLD
Z_Shift_L_Doxo_EG
Z_Im_L_Doxo_EG
